# Paper-based microfluidics for wearable soft bioelectronics

**DOI:** 10.1039/d5lc00754b

**Published:** 2026-01-07

**Authors:** Feng Zhang, Ganggang Zhao, Qunle Ouyang, Sicheng Chen, Zheng Yan

**Affiliations:** a Department of Chemical and Biomedical Engineering, University of Missouri Columbia MO USA yanzheng@missouri.edu; b Department of Mechanical and Aerospace Engineering, University of Missouri Columbia MO USA; c NextGen Precision Health, University of Missouri Columbia MO USA

## Abstract

Wearable biosensing technologies are transforming healthcare by enabling continuous, real-time monitoring of physiological states at the point of care. Flexible microfluidics, particularly paper-based microfluidics, serve as critical interfaces between the body and soft electronics, enabling precise, capillary-driven, and non-invasive biofluid handling for real-time and clinically informative diagnostics. In this review, we discuss the fundamentals of paper-based microfluidics, highlighting critical considerations in material design, structural regulation, and interface engineering for precise capillary flow manipulation. We revisit fabrication techniques and key milestones in developing paper-based microfluidic devices, emphasizing innovative on-skin applications for wearable biofluid sampling, biosensing, and disease diagnostics. Finally, we outline persistent challenges that need to be addressed in the clinical translation of paper-based microfluidics for wearable healthcare and discuss future perspectives, including advances in paper materials engineering, integration with machine learning algorithms, and Internet-of-Things, to enable the next-generation personalized wearable healthcare solutions.

## Abstract 2

We provide a comprehensive review of state-of-the-art paper-based microfluidic devices for wearable soft bioelectronics, emphasizing innovative materials and device designs that enable on-skin biofluid sampling, biosensing, and disease diagnostics.

## Graphical abstract

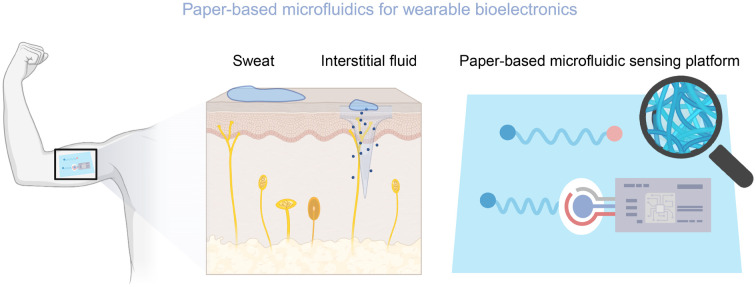

## Introduction

Wearable biosensing technologies are poised to revolutionize healthcare monitoring and personalized medicine by enabling real-time, non-invasive, and continuous tracking of vital biophysical and biochemical signals.^1–6^ These advanced systems facilitate the diagnosis and management of infectious diseases and various health conditions directly at the point of care.^7,8^ By continuously analyzing biofluids such as sweat, saliva, tears, interstitial fluid, and wound exudate, wearable sensors can provide clinically relevant insights essential for managing diverse health conditions, such as metabolic disorders and infectious diseases.^9–11^

Microfluidics serve as the foundation of on-skin wearable systems, enabling precise handling and analysis of biofluid volumes ranging from nanoliters to microliters within microscale channels.^12–14^ Originally adapted from semiconductor microfabrication techniques, recent advances in materials science have facilitated the creation of flexible and stretchable microfluidic platforms, termed “microelastofluidics”.^15,16^ These systems offer passive while elegant solutions for continuous biofluid extraction and transport, eliminating the necessity for external pumps or pressure-control mechanisms.^17–19^ Microfluidics significantly enhance the functionality of wearable sensors by enabling precise flow regulation, efficient analyte collection, effective sample separation, targeted delivery to sensing sites, and real-time biochemical analysis.^20–22^ Among the various materials explored for developing flexible microfluidic platforms, paper-based microfluidic devices have emerged as a highly compelling and sustainable solution.^23–25^ Paper, composed of entangled cellulose fibers, offers multiple functional advantages, including global availability, low cost, and suitability for disposable diagnostics, particularly in resource-limited settings.^25–27^ Its inherent porosity facilitates spontaneous fluid wicking *via* capillary action, eliminating the need for powered fluid transport mechanisms.^28–30^ Additionally, paper's intrinsic flexibility and patterning capabilities enable microfluidic devices to comfortably conform to skin contours while preserving functionality.^31–33^ The breathable and biocompatible properties of cellulose minimize skin irritation and enhance long-term wearability.^34,35^ Furthermore, the porous structure of paper allows for *in situ* reagent storage, simplifies sample preparation, and supports various detection modalities, including colorimetric, optical, and electrochemical assays.^36–38^ Recent advances in electrofluidic integration, including the embedding of conductive polymers and laser-induced graphene, have transformed paper-based microfluidic devices into multifunctional platforms capable of serving as fluidic channels, electrodes, and interconnects simultaneously.^39–41^ These innovations facilitate the integration of Bluetooth or near-field communication (NFC) capabilities and enable quantitative, multiplexed, point-of-care diagnostics.^42–44^ Moreover, the simplicity of fabrication processes such as wax printing, inkjet printing, screen printing, and laser cutting further promotes rapid prototyping and scalable manufacturing of paper-based microfluidic devices and systems.^45–48^ These integrated advantages establish paper-based microfluidics as a leading innovation in wearable diagnostics, which is significant in the development of telehealthcare and telemedicine.^49–53^ To date, paper-based microfluidic devices have exhibited a wide range of applications, including nutrient assessment, metabolite tracking, pathogen detection and continuous monitoring of low concentration inflammatory biomarkers and hormones.^30,54–59^ These biomarkers are critical indicators of conditions such as diabetes, cardiovascular disease and renal dysfunction.^14,60^

**Fig. 1:**
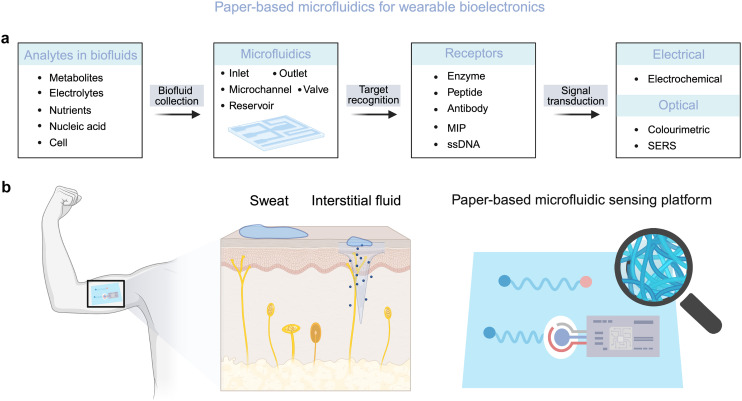
Paper-based microfluidics for wearable biomedical applications. (a) Flexible paper-based capillary microfluidic platforms enable biofluid collection, target recognition, and signal transduction through integration with soft microelectronics. (b) Wearable paper-based microfluidic sensing systems designed for the real-time analysis of sweat and interstitial fluid.

This review aims to provide a rational guideline for the fabrication of paper-based microfluidics tailored for wearable soft bioelectronics. We discuss strategies in materials design, structural engineering, and interface modulation of paper materials that enable precise control of capillary-driven fluid handling. Furthermore, we review current approaches for developing wearable paper-based microfluidic devices for non-invasive biofluid sampling, biosensing, and disease diagnostics. Finally, we offer a perspective on current bottlenecks, ongoing challenges, and future opportunities for paper-based wearable microfluidics, highlighting their potential to advance personalized and preventive medicine by bringing laboratory-grade diagnostics closer to individuals than ever before (Fig. 1).

## Fundamentals of paper-based microfluidics

### Material properties of paper for microfluidic functionality

**Fig. 2:**
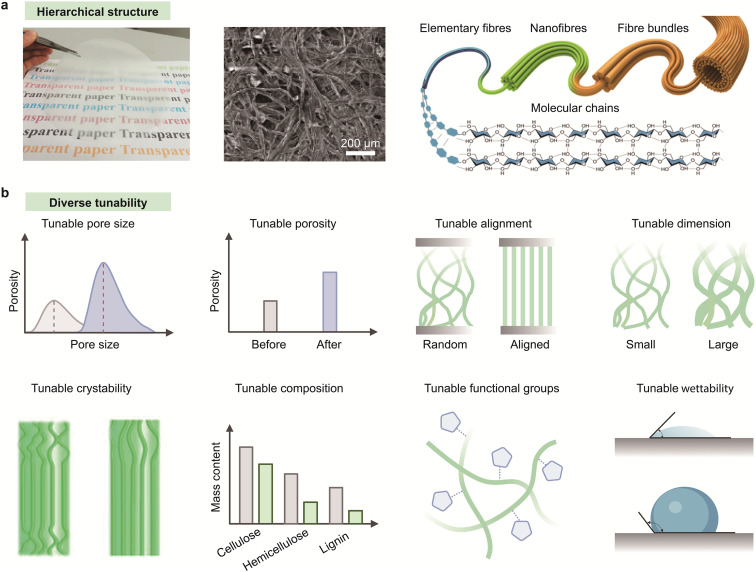
Paper-based materials and their structural tunability. (a) Hierarchical fibrous structure of cellulose-based paper. (b) Diverse structural tunability of paper-based materials including regulation over pore size, porosity, fiber alignment, dimensionality, crystallinity, chemical composition, surface molecular structure, and wettability. Reproduced from ref. 25 with permission from Springer Nature, copyright 2020. Reproduced from ref. 33 with permission from ACS Publications, copyright 2014.

Paper, also known as cellulose film, assembled by fibrillated cellulose, has emerged as a compelling substrate for microfluidic devices because of its integrated advantages of intrinsic capillary action, hierarchical architecture, high porosity, mechanical flexibility, and ease of chemical modification (Fig. 2a).^13,34^ Notably, the naturally interconnected fibrous network of paper enables passive, pump-free fluid transport through enhanced capillary forces, making it particularly promising for point-of-care diagnostics and wearable biosensing applications.^58,61,62^Table 1 summarizes the typical materials used to fabricate microfluidic devices.^149–174^

**Table 1:** Summary of representative materials used in microfluidic devices

Material category	Representative materials	Advantages	Challenges	Ref.
Inorganic-based	Si	High strength; precise platforms for high-resolution micro–nano fabrication; long term durability	High cost; complex fabrication; limited stretchability; cleanroom requirement	149
	SiO_2_			150
	SiC			151
	Ti			152
	Steel			153
Polymer-based	Polydimethylsiloxane	Highly flexible; stretchable; mechanical stability; chemical resistant; compatible with microfabrication	Requires external pumping; residual stress; limited permeability; less sustainable	154
	Ecoflex			155
	Polyethylene terephthalate			156
	Polyimide			157
	Parylene C			158
	Polypropylene			159
	Polycarbonate			160
	Poly(methyl methacrylate)			161
	PTFE			162
	TPU			163
	PLGA			164
	PLA			165
Hydrogel-based	Gelatin	Soft; hydrated; biocompatible; mechanical compliance	Swelling; dehydration over time; mechanical instability	166
	Alginate			167
	PEG			168
	PAA			169
	PVA			170
Textile-based	Cotton	Breathable; high flexibility; conformal contact with skin	Limited control over fluid handling; contamination from sweat/salt; challenges in integration with rigid electronics	171
	Nylon			172
	PET			173
Paper-based	Cellulose	Biodegradable; low-cost; lightweight; capillary-driven fluidics; easy surface modification	Sensitive to moisture and contamination (sweat, dust); limited mechanical durability; limited long-term stability	174

Unlike conventional polymeric microfluidic substrates, which often require external actuation and complex fabrication processes, paper inherently supports spontaneous, programmable fluid transport *via* capillary action with minimal infrastructure.^13,63–65^ Capillary flow in paper is dictated by its microstructural properties, including pore size, porosity, fiber orientation, and tortuosity (Fig. 2b), allowing application-specific fluidic pathways to be precisely engineered for controlled flow rates, analyte retention, and fluid distribution.^66,67^ Furthermore, the surface of paper substrates can be easily functionalized to tune wettability and direct fluid flow. Functionalization techniques such as wax printing, inkjet patterning, plasma treatment, and silanization have also been widely employed to define hydrophobic barriers and channel geometries with high spatial resolution.^60,68–72^ These modification strategies enable valveless fluid control and seamless integration with multiplexed sensing assays. More recently, laser-induced graphenization has emerged as a powerful approach to directly pattern conductive graphene electrodes onto the paper matrix, facilitating *in situ* electrochemical sensing without compromising capillary performance.^73,74^

When it comes to wearable applications, paper's exceptional conformability and breathability are critical attributes for effective on-skin biofluid management. Its inherent lightweight and flexibility enable intimate, stable contact with the skin, allowing for continuous and reliable fluid collection and signal acquisition without compromising user comfort.^75^ Additionally, the biodegradability and low cost of paper-based materials align with the global demand for sustainable, disposable diagnostic platforms.^76^ Advances in cellulose processing have broadened the material palette beyond conventional paper. For instance, top-down delignified wood structures and bottom-up nanocellulose films both demonstrate enhanced mechanical tunability, increased specific surface area, and anisotropic fluid flow.^77–79^ These engineered cellulose-based materials present new opportunities for regulating analytes, transporting biomolecules, and seamlessly integrating with bioelectronic components, thereby paving new avenues for paper-based capillary systems for next-generation microfluidic and wearable bioelectronic technologies.^80–84^

### Mechanisms, functions and configurations of paper-based microfluidic devices

**Fig. 3:**
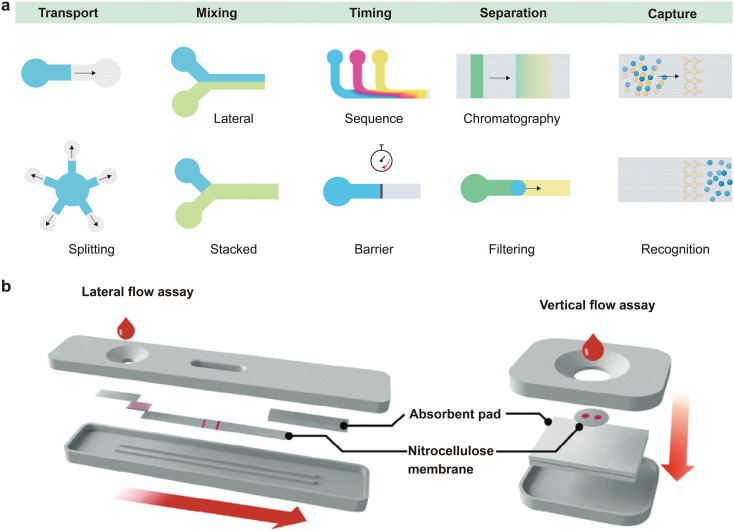
Functions and configurations of paper-based microfluidic devices. (a) Capillary flow-enabled fluidic functions in paper-based microfluidics, including transport, mixing, timing, separation, and analyte capture. (b) Typical configuration of commercial paper-based microfluidic assays used for point-of-care diagnostics. Reproduced from ref. 37 with permission from Wiley-VCH, copyright 2024.

In paper-based microfluidic systems, fluid motion is passively manipulated by leveraging the intrinsic capillarity of the paper's porous network, without the need for external pumps, valves or power sources.^85,86^ This mode of fluid manipulation is essential for the simplicity and accessibility of microfluidic paper-based analytical devices (μPADs), as well as for integrating them into complex wearable bioelectronic systems.^87,88^ The microstructural architecture of the paper finely tunes the fundamental principles of capillary flow, enabling a broad range of essential fluidic operations, such as transport, mixing, timing, separation, and selective analyte capture, for advanced analytical and diagnostic applications (Fig. 3a and b).^89^

Typically, in paper-based microfluidics, the primary mechanism driving fluid movement is capillary action, a phenomenon governed by the interplay between interfacial tension and viscous forces. In porous materials such as cellulose paper, fluid flow generally occurs under laminar (Stokes) flow conditions, which are characterized by low Reynolds numbers. The velocity of the advancing fluid front can be described by Darcy's law, and under idealized conditions it is well approximated by the Washburn equation:^90–93^
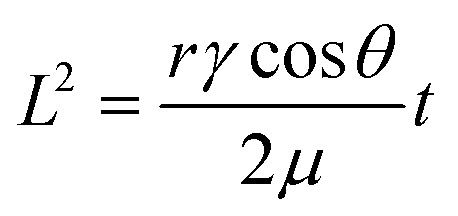
where *L* represents the distance travelled by the fluid front at time *t*, *r* is the effective pore radius, *γ* denotes the surface tension, *θ* is the contact angle, and *μ* is the dynamic viscosity of the liquid. This equation reveals that the wicking rate can be enhanced by increasing surface energy and pore size.^94^ Moreover, a hydrophilic surface with a contact angle of lower than 90° is necessary to generate positive capillary driving pressure.^73,95–98^ In more complex microfluidic systems involving heterogeneous materials or variable channel geometries, fluid dynamics can be modelled in a way that is analogous to electrical circuits, where capillary pressure is like voltage and hydrodynamic resistance corresponds to electrical resistance.^99^ This analogy facilitates the rational design of fluidic networks, enabling biofluids to be directed along paths of least hydrodynamic resistance.

Engineered paper-based microfluidic platforms with defined structural channels can support a wide variety of fluidic manipulations, such as transport, mixing, timing, sequencing, separation and analyte capture.^45^ As illustrated in Fig. 3a: (i) transport: fluids are directed laterally, or vertically in three-dimensional architectures, between stacked layers *via* hydrophilic channels bounded by hydrophobic barriers.^100,101^ An absorbent pad is often used as a capillary pump or sink to create a sustained pressure gradient and maintain directional flow. (ii) Mixing: efficient mixing of reagents, which is typically difficult under low-Reynolds-number conditions characteristic of capillary-driven flow, is facilitated through geometric modifications, such as stacked 3D junctions that increase the interfacial contact area, or patterned structures that promote advective mixing.^102,103^ (iii) Timing and sequencing: temporal control over reagent delivery can be achieved using passive strategies, such as varying channel lengths to introduce flow delays, incorporating dissolvable barriers or integrating passive valve mechanisms.^104^ Capillary bursting valves (CBVs) and hydrophobic valves generate pressure thresholds that temporarily halt flow until a defined capillary pressure is reached. This enables sequential operations, which are essential for multi-step assays such as enzyme-linked immunosorbent assay (ELISA).^105,106^ (iv) Separation: μPADs can separate sample components based on physical or chemical principles, such as chromatographic partitioning, size-exclusion filtration or charge-mediated diffusion. These mechanisms enable operations such as plasma extraction from whole blood, nucleic acid purification, and microorganism isolation.^107–109^ (v) Capture and recognition: target-specific capture is achieved by immobilizing biorecognition elements, such as antibodies, aptamers or nucleic acids, onto a porous matrix such as nitrocellulose paper.^110^ Capillary flow transports the sample through the capture zone, delivering the target analyte to the immobilized receptors and removing any unbound species. This principle underlies both lateral and vertical flow immunoassays.

## Design and fabrication of paper-based microfluidic devices

Paper-based microfluidic devices leverage the inherent structural and chemical features of cellulose fibrils to serve as versatile platforms for real-time biofluid sampling, biosensing, diagnostics.^111,112^ Intuitively, the functionality of paper-based microfluidic devices depends on the precise definition of fluidic pathways, reaction chambers, and sensing zones within the porous paper matrix.^37^ To date, a broad spectrum of various fabrication strategies has been established to pattern microfluidic architectures onto mechanical compliant paper substrates.^113^ Each approach entails specific trade-offs in channel resolution, scalability, cost, and compatibility with functional materials.^50,55,114,115^ In the following section, we critically review four principal fabrication modalities including printing, laser processing, photolithography, and other emerging techniques for fabricating paper-based microfluidic devices.

### Printing

Printing-based techniques (*e.g.*, wax printing, inkjet printing, and screen printing) represent the most accessible and scalable approaches for the fabrication of paper-based microfluidic devices.^116^ Notably, hydrophobic barriers could be patterned on various substrates by high-throughput, mask-free printing to delineate microfluidic channels.^117^ Moreover, various functional components, for example, electric traces, electrodes, and sensing interfaces could be well-deposited onto paper substrates to enable the integration of multifunctional and multimodal devices.^118^

Wax printing is a straightforward method to fabricate hydrophobic barriers or walls on paper substrates. Typically, solid wax is deposited onto paper followed by thermal treatment, thus enabling the lateral and vertical diffusion of melted wax throughout the porous matrix.^119^ This process effectively establishes hydrophobic boundaries that could guide capillary flow and is particularly adapted to rapid prototyping and scaleable production of low-cost, disposable diagnostic devices.^120^ Nevertheless, the spatial resolution of wax printing is limited due to uncontrolled wax diffusion, posing challenges for the development of miniaturized and highly integrated devices or systems.

**Fig. 4:**
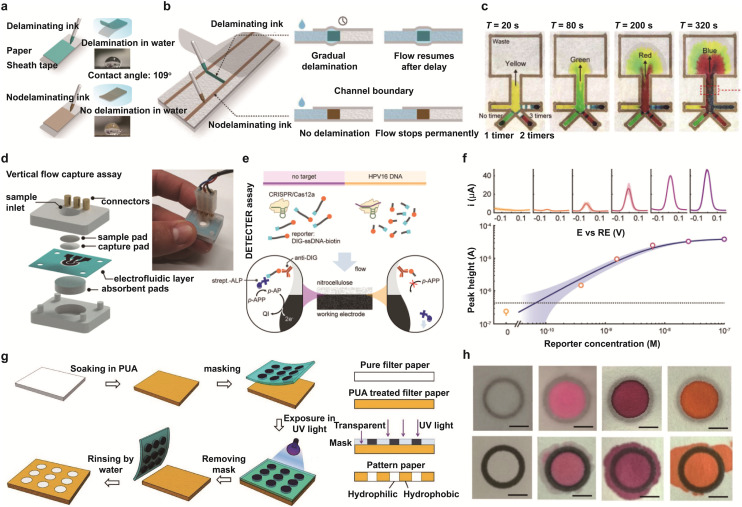
Design and fabrication of paper-based microfluidic devices. (a) Capillary flow modulation on paper through pattern imprinting. (b) Illustration of a paper-tape timing system using water-insoluble ink. Delaminating ink enables timed resumption of flow *via* void formation at the interface, whereas non-delaminating hydrophobic ink creates permanent flow barriers. (c) Time-lapse images of a test platform with four dye solutions introduced from separate inlets. Differential delays in capillary flow, achieved by varying the number of drawn timers, enable sequential delivery. Reproduced from ref. 143 with permission from American Association for the Advancement of Science, copyright 2022. (d) Schematic of a multilayer vertical flow device consisting of a capture pad, a laser-induced graphene (LIG) electrode and an absorbent pad. (e) Electrochemical detection assay based on target capture on a porous electrode. (f) Square wave voltammograms for buffer samples with varying target concentrations and the corresponding Langmuir adsorption fit. Reproduced from ref. 130 with permission from Wiley-VCH, copyright 2023. (g) Fabrication of paper-based microfluidic channels using polyurethane acrylate (PUA)-based UV patterning. (h) Comparison of surfactant resistance between PUA-based barriers (top) and wax-printed barriers (bottom). Reproduced from ref. 69 with permission from Elsevier, copyright 2020.

The inkjet printing method has garnered great attention because of its ultrahigh resolution and extensive design flexibility. This maskless and digitally controlled deposition method enables rapid prototyping and direct integration of various functional materials through the ejection of picolitre-scale droplets of specialized inks.^121^ Of note, complex multilayer architectures can be fabricated from functional inks loaded with materials such as silver nanoparticles, nanowires, graphene, and immobilized enzymes.^122,123^ Recently, a technique was reported to control capillary flow on paper substrates *via* precisely imprinting blockers or timers on the fluidic pathways (Fig. 4a).^60^ Notably, water-soluble inks were printed as “roadblocks” on the channel and the gradual formation of voids between wetted paper and a polymer sheath were employed to fabricate “timers” (Fig. 4b). This interesting design could enable multiple liquid to be guided to stepwise chemical reactions with well-defined sequence. A device with four inlet branches and a single channel outlet was fabricated to coordinate capillary flow streams (Fig. 4c). Moreover, the flow in each branch could be differentially delayed by varying timer numbers. Nevertheless, the development of various inks with printability on paper substrates is still limited and the stability of ink on paper substrates decreases over an extended period window. With the expansion of available inks with robustness, paper-based microfluidic devices could be further used in advanced point-of-care diagnostic tools such as protein assays.

Screen printing and flexographic printing are alternative methods for fabricating paper-based microfluidic devices, especially for large-scale and roll-to-roll production.^124^ Screen printing forces viscous inks through a patterned mesh, thereby enabling rapid electrode fabrication and the formation of well-defined hydrophobic boundaries.^125^ Flexographic printing is a high-speed, roll-to-roll process that could support continuous, high-throughput patterning of channel walls at an industrial scale.^126^ Despite the advantages of large-scale manufacturing, it is less amenable to rapid design iteration because the fabrication of new screens or printing plates constitutes a major bottleneck to customization.

### Laser engraving

Laser engraving is a widely used, mask-free technique for creating microfluidic structures, involving the selective ablation of material from paper or laminated films.^127^ This digital method enables precise control over channel geometries and supports the construction of multilayer devices by laminating patterned sheets together.^128^ Automated computer-aided design and manufacturing (CAD/CAM) workflows enable the high-resolution patterning of microfluidic channels. However, thermal damage remains a significant challenge. The heat generated during laser ablation can cause the pore structure to collapse locally and deposit hydrophobic residues along the cut edges. Both of these issues seriously hinder capillary-driven wicking.^129^ Despite these limitations, laser processing is widely used for the rapid prototyping and scalable fabrication of complex, multilayer microfluidic devices. Recently, a paper-based, laser-pyrolyzed electrofluidic platform was developed as an electrochemical system for capillary-driven diagnostic assays (Fig. 4d).^130^ With integrated wax lamination, both fluidic pathways and isolated electrode zones were clearly defined. A vertical capture assay was ingeniously assembled, including a buffer pad, a nitrocellulose capture layer, an electrofluidic layer, an antibody-functionalized nitrocellulose pad, a reservoir, a sample injection port and connectors. Capillary flow between the reservoir and absorbent pad enabled fluid movement both laterally along the channel and across the laser-induced graphene (LIG) zones. This setup enables the rapid and continuous analysis of up to 24 samples in 20 minutes, with less than 5 μL required per sample. The paper-based electrofluidic assay demonstrated an ultralow detection limit of 67 pM and a quantification range extending up to 60 nM for CRISPR reporter analysis (Fig. 4e and f).

### Photolithography

Photolithography is widely regarded as the gold-standard technique for achieving high-resolution patterning on a variety of substrates. The process typically involves impregnating paper with a liquid photoresist, followed by exposing it to patterned ultraviolet (UV) light through a photomask.^131,132^ Subsequent curing steps generate well-defined contrasts in the distribution of polymers and surface energy, resulting in precise hydrophilic channels—often with feature resolutions below 200 μm—bounded by hydrophobic barriers. This high-resolution capability allows sophisticated microfluidic components, such as valves, mixers and discrete reagent zones, to be integrated within compact device architectures.

Furthermore, the surface chemistry of commonly used photoresists such as SU-8 can be easily modified to enable the covalent immobilisation of biomolecules, thereby enhancing target-specific analyte capture and improving reaction specificity. Various microfluidic polymer chips have been fabricated using polyurethane acrylate (PUA). Notably, water-based PUA has been developed to pattern hydrophobic barriers on paper substrates, effectively preventing boundary degradation when exposed to organic solvents (Fig. 4g).^69^ Unlike wax-based barriers, PUA-defined boundaries remain intact and are not breached, even in the presence of high-concentration surfactant solutions (Fig. 4h).

Despite these advantages, photolithography requires specialised materials (photoresists), UV exposure equipment and a cleanroom environment, which limits accessibility and increases fabrication costs.^133^ Additionally, residual photoresists may autofluoresce or absorb light at analytical wavelengths, potentially interfering with optical detection methods. This further necessitates careful material selection and rigorous process optimisation, particularly for colorimetric or fluorescence-based assays.

### Others

Several alternative and hybrid fabrication methods have expanded the design landscape of μPADs beyond conventional techniques. Plasma treatment allows for the selective modification of surfaces by exposing pretreated paper to plasma through a patterned mask. This technique alters surface energy and wettability without the need for additional materials, making it ideal for patterning large-area devices or controlling fluid flow in complex geometries.^134^ Chemical etching, such as inkjet-based etching of polystyrene-coated paper, converts hydrophobic surfaces into high-fidelity hydrophilic patterns.^135^ This approach has proven effective in multi-analyte detection systems while maintaining compatibility with colorimetric and enzymatic assays. Stamping and embossing methods offer low-cost fabrication using wax- or ink-loaded stamps, providing rapid prototyping capabilities while achieving high resolution is a challenging issue.^136,137^ Hybrid approaches, such as combining wax printing with laser cutting or inkjet etching, enable the fabrication of custom paper-based microfluidic devices that optimize fluid control, signal generation and mechanical durability.^138^ These strategies are increasingly being employed to integrate sensing elements, flow regulators and signal transduction modules within unified, paper-based platforms.

Overall, these fabrication strategies provide a versatile toolkit for transferring functional microfluidic designs onto paper substrates. The optimal method depends on application-specific parameters, such as resolution requirements, material compatibility, production cost and scalability. In the case of wearable bioelectronic systems in particular, the combination of printing and laser-based techniques shows great promise, as it enables the development of flexible, disposable and multifunctional devices for continuous wearable health monitoring and point-of-care diagnosis. For the mass-production of wearable, paper-based microfluidic devices, it is important to balance scalability and cost-effectiveness with the integration of multifunctional components. Printing methods, including wax printing, inkjet printing and flexographic printing, are the most suitable for large-scale production. These methods are cost-efficient and easily scalable, making them ideal for manufacturing flexible, disposable devices at a large scale. Wax printing is particularly effective at creating hydrophobic barriers on paper, enabling capillary flow for fluid manipulation, while inkjet printing enables the precise deposition of functional materials, such as electrodes and sensors, directly onto the substrate. Flexographic printing, with its roll-to-roll capability, further enhances scalability and production speed, making it an excellent option for continuous, large-scale manufacturing. In contrast, laser-based techniques, such as laser engraving, offer high precision for intricate designs and multilayered structures. However, they are less cost-effective and scalable compared to printing methods, making them less suitable for mass production. Although laser engraving is ideal for prototyping or producing complex, high-performance devices, it is not cost-efficient for large-scale production due to higher costs, energy consumption, and limited scalability. Therefore, the optimal approach for mass-producing wearable paper-based microfluidic devices is to integrate standard production printing techniques with laser engraving for advanced features, thereby striking a balance between functionality and manufacturing efficiency.

## Applications of paper-based microfluidics in wearable soft bioelectronics

### On-skin wearable biofluid sampling

Non-invasive sampling of biofluids such as sweat, interstitial fluid (ISF), saliva, and wound exudate is critical for developing next-generation wearable healthcare monitoring systems.^14^ These biofluids contain a rich array of biomarkers, including electrolytes, metabolites, hormones, cytokines, and nucleic acids, which reflect systemic physiology, infection status, and disease progression.^16,22,27^ Real-time access to the dynamic fluctuations of these analytes enables continuous health tracking, early disease detection, and personalized therapeutic interventions.

However, achieving reliable and continuous sampling of epidermally accessible biofluids remains technically challenging. Sweat secretion is intermittent and varies significantly between individuals. ISF is located several hundred micrometers beneath the skin surface, necessitating minimally invasive extraction techniques. Saliva is highly susceptible to contamination from food and oral microbiota, while wound exudate is often heterogeneous in both composition and volume, particularly across different stages of inflammation. Consequently, the ultralow and fluctuating secretion rates of these fluids necessitate the development of sampling systems that are highly sensitive and passively driven, thus minimizing reliance on external power sources.

Wearable microfluidic platforms offer robust capabilities for the continuous collection and management of biofluids. These ingeniously engineered systems incorporate microchannels, valves, reservoirs and selectively permeable interfaces into skin-conforming architectures that wick fluids passively *via* capillary action. This eliminates the need for external pumps or power-intensive actuators.^139,140^ For example, epidermal sweat patches containing microfluidic channels with precisely aligned inlets and outlets have successfully directed and analysed perspiration during rest and physical activity. Similarly, microneedle-integrated devices provide minimally invasive access to interstitial fluid (ISF) while maintaining skin integrity for long-term sampling.

Among various available microfluidic strategies, paper-based microfluidic (PBM) systems offer unique advantages in terms of capillary performance, ease of fabrication, and user comfort. Constructed from soft, flexible, porous cellulose substrates, PBM devices utilize the intrinsic capillarity of fibrous networks to autonomously drive fluid flow without external actuation. Furthermore, their ultrathin, lightweight and breathable properties minimize skin irritation, supporting long-term wearability, particularly in ambulatory or continuous monitoring scenarios. Table 2 summarizes current point-of-care paper-based microfluidic biosensors, categorized into qualitative and quantitative biosensing. Qualitative devices rely on visually interpretable signals (*e.g.*, colorimetric or lateral-flow responses), while quantitative devices incorporate electrochemical or optical transduction to provide precise signal outputs. This highlights the trade-off between simplicity and precision, along with the technological evolution toward continuous, accurate, and reliable multi-analyte wearable biosensing and diagnostics.^143,175–188^

**Table 2:** Overview of recent advances in paper-based microfluidic technologies and their biosensing applications

Category	Sensing method	Analyte and corresponding LOD	Fabrication method	Features	Ref.
Qualitative biosensing	Colorimetric	Myeloperoxidase	Wax printing	Detecting wound infection based on the difference of color intensity	175
	Colorimetric	Human neutrophil elastase	Wax printing	Visible colorimetric reaction between the paper substrate and analytes	176
	Colorimetric	β-Lactamase	Surface coating	Paper-based bacterial resistance colorimetric card	177
	Colorimetric	pH, glucose	Lamination, laser cutting	Reduced sweat evaporation	178
Quantitative biosensing	Chronoamperometry (CA)	Glucose: 59.5 μM	Wax printing, stencil printing	Low LOD	179
	CA	Lactate: 0.36 mM	Photolithography, screen-printing	The first paper-based microfluidic electrochemical device	180
	CA	Adenosine: 11.8 μM	Wax printing, screen printing, origami	Self-powered microfluidic sensor	181
	CA	Paracetamol: 25 μmol L^−1^	Wax printing, sputtering	Separation of target analytes by the paper microchannel	182
	CA	Ascorbic acid: 30 μM	Wax printing, pencil drawing	Separation of target analytes by the paper microchannel	183
	CA	β-Hydroxybutyrate	Inkjet printing, laser cutting	Simultaneous detection of glucose and β-HB in sweat	142
	Differential pulse voltammetry (DPV)	AFP: 0.01 ng mL^−1^	Wax printing, screen printing, origami	Highly integrated devices for cancer diagnosis	184
	DPV	Melamine: 1.0 μM	Ink writing	Ease-of-fabrication by writing electrodes on paper	185
	Square wave voltammetry (SWV)	Cd^2+^: 11 ppb	Wax printing, screen printing, cutting	Capable of detecting mud-spiked samples	186
	SWV	Pb^2+^: 1.8 μg L^−1^	Screen printing, cutting, stacking	Modifier-free electrodes	187
	Anodic stripping voltammetry (ASV)	Pb^2+^: 1.0 ppb	Photolithography, screen-printing	Enhanced sensitivity with fluidic analyte	188
	Plasmonic	Uric acid	Mechanical cutting	Quantification of uric acid in sweat at physiological and pathological concentrations	143

**Fig. 5:**
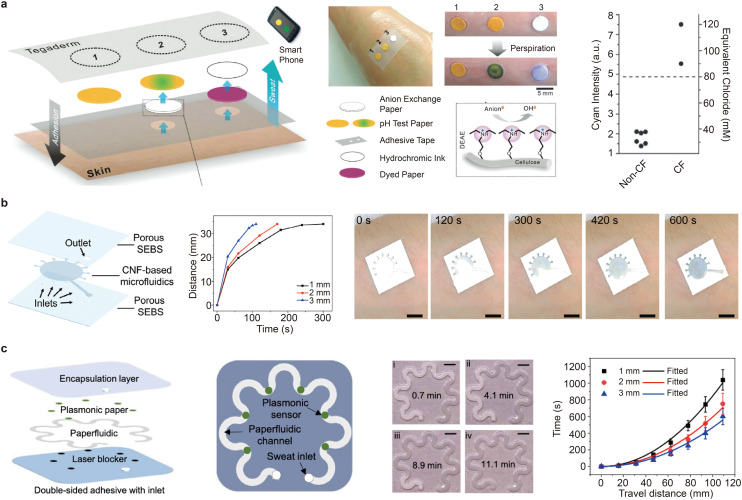
Paper-based microfluidic devices for on-skin wearable biofluid sampling. (a) A paper-based skin patch designed for the vertical transport of sweat *via* capillary-driven flow. Reproduced from ref. 141 with permission from Royal Society of Chemistry, copyright 2015. (b) CNF-based paper microfluidics for continuous wearable sweat monitoring. Reproduced from ref. 142 with permission from Wiley-VCH, copyright 2025. (c) Multilayer paperfluidic device that integrates stacked functional layers and serpentine microchannels for efficient sweat routing on skin. Reproduced from ref. 143 with permission from American Association for the Advancement of Science, copyright 2022.

Notably, paper substrates facilitate a variety of strategies for controlling capillary fluid, such as spatial flow confinement through hydrophobic–hydrophilic patterning and temporal flow regulation using dissolvable valves or delaminating timers.^76^ Integrating hydrogel coatings or porous absorbent layers can further modulate fluid uptake under low-secretion conditions, minimizing sample evaporation and backflow.^141^ For instance, a paper-based, wearable diagnostic system incorporating absorbent paper, anion-exchange paper and pH test strips has been reported (Fig. 5a). Importantly, paper with a smaller pore size and a faster capillary wicking rate enables rapid sweat sampling, thereby reducing the time and physical effort required for sweat extraction.

More recently, a cellulose nanofibril (CNF)-based wearable microfluidic system was developed on a porous elastomeric substrate (Fig. 5b).^142^ Unlike conventional hollow microfluidic channels fabricated on flexible matrices, the CNF interface featured enhanced porosity and capillary wicking performance, facilitating efficient sweat transport. The integrated microfluidic system had a total thickness of 200 μm. The liquid-wicking kinetics of the CNF channels could be precisely tuned by adjusting their width and thickness. In an on-body sweat extraction experiment, the sensing chamber was completely filled within seven minutes following iontophoresis-induced sweat stimulation. Maintaining the structural integrity of paper-based microfluidic channels for long-term wearable biofluid sampling remains a significant challenge. To address this issue, a serpentine structural design was adopted in the fabrication of a paper-based microfluidic system referred to as a ‘paperfluidic’ device (Fig. 5c).^143^ This wearable system comprises multiple functional layers, including a laser blocker, an adhesive layer, a paper-based microfluidic channel, plasmonic sensors and an encapsulation layer. The serpentine layout of the microfluidic channels enabled the device to accommodate skin deformation with minimal stress concentration and structural degradation. Quantitative continuous flow testing showed that the distance travelled by liquid increased proportionally with flow time. Furthermore, both travel time and distance could be easily adjusted by changing the width of the channels in the ‘paperfluidic’ device.

Overall, from a translational perspective, PBM devices are inherently low-cost and compatible with various scalable manufacturing techniques, such as wax printing, inkjet deposition and laser pyrolysis. These combined advantages establish PBM systems as a promising platform for epidermal biofluid sampling and handling, while supporting cost-effective production and enabling real-time, point-of-need diagnostics in everyday settings.

### On-skin wearable biosensing

On-skin microfluidic biosensing devices integrate sensor electrodes, microfluidics and soft, skin-conforming substrates to enable the real-time, non-invasive monitoring of biochemical and physiological signals directly from the epidermis.^11,73^ These systems provide clinically meaningful insights into individual health, infection status and disease progression. Paper-based capillary microfluidics play a critical role in not only biofluid collection and routing, but also directing analytes to sensing zones while minimising sample crosstalk.^14^ Layered channel designs, evaporative reservoirs and burst valves enable precise temporal control of biofluid delivery, supporting chrono-sampling and reducing analyte dilution. These fluidic architectures on paper substrates allow high-fidelity signals to be generated, particularly under dynamic conditions characterized by fluctuating sweat rates or biomarker concentrations. A variety of sensing modalities, including plasmonic, colorimetric, electrochemical and optical techniques, have been successfully integrated with microfluidic modules to create wearable, skin-interfaced biosensing systems.^28,38^ The unique properties of paper have notably redefined conventional electroanalytical approaches and inspired novel sensing strategies at the intersection of capillary-driven microfluidics and wearable bioelectronics.

Paper-based plasmonic sensors that integrate surface-enhanced Raman spectroscopy (SERS) with capillary microfluidics can capture, detect and quantify a wide range of analytes, including metabolites, bacteria and macromolecules, without the need for labels.^144^ Notably, the Raman bands of the analytes—which arise from their characteristic rotational and vibrational modes—provide precise molecular ‘fingerprints’ that enable accurate identification. Recently, a wearable, paper-based, microfluidic plasmonic platform, known as a ‘paperfluidic’ system, was developed for the continuous, reliable and *in situ* extraction of sweat (Fig. 5a).^143^ In this system, plasmonic sensors are positioned at various points along the capillary channel to quantify analyte concentrations in sweat collected at different time intervals. An encapsulation layer made of polydimethylsiloxane (PDMS), which is optically transparent and produces well-defined Raman bands, was integrated as a reference element to enable accurate quantification of sweat analytes. This integrated paperfluidic system was successfully interfaced with human skin to monitor sweat chemistry in real time. The quantified uric acid concentration in sweat was 28 μM, which aligns well with values typically observed in healthy individuals.

The rapidly growing demand for the quantitative, real-time detection of key biomarkers associated with metabolic regulation, fatigue monitoring and chronic disease management has fuelled recent momentum in the development of precise biosignal extraction technologies.^145^ Electrochemical sensors, particularly those based on enzymatic or redox reactions, are widely used in wearable soft bioelectronic systems thanks to their high sensitivity, compact size and compatibility with low-power electronics. In this context, electrochemical paper-based microfluidics further enhance analytical performance by enabling spatially resolved electron transfer and multiplexed detection, as they integrate conductive traces and electrochemically active electrodes directly into cellulose substrates.^146,147^ However, fabricating electrodes on paper remains challenging due to the high surface roughness and heterogeneous architecture of cellulose fiber networks. Various electrode deposition strategies have therefore been explored to construct paper-based electrochemical biosensors within microfluidic architectures, including metal sputter coating, screen printing, and manual methods such as pencil drawing.^148^

**Fig. 6:**
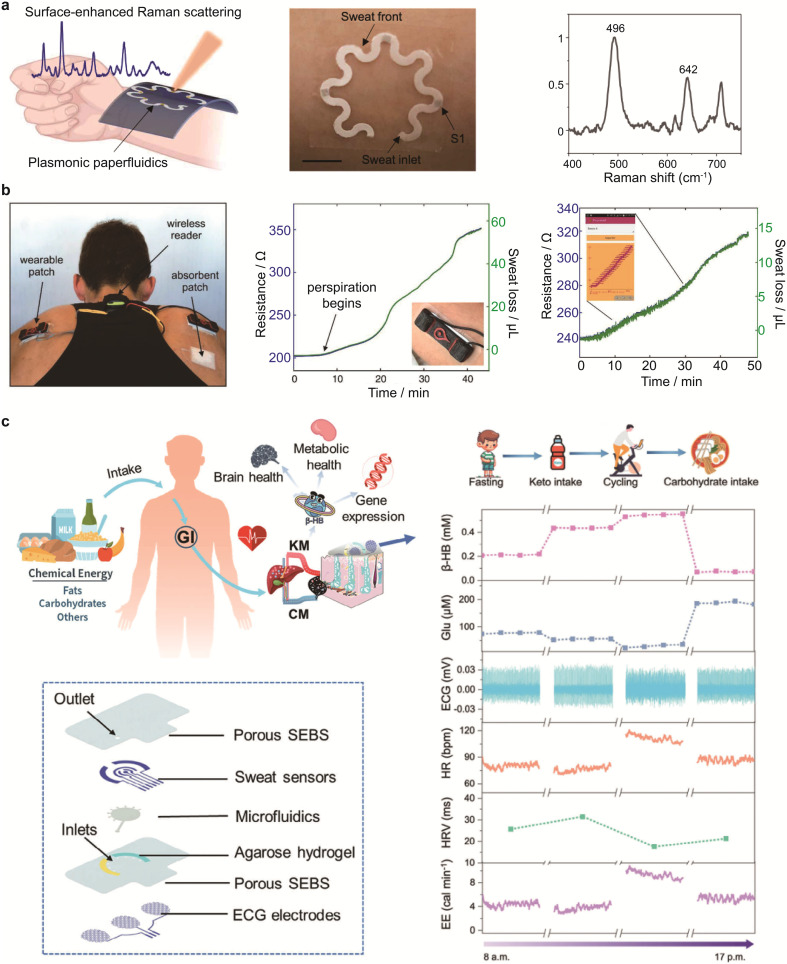
Wearable paper-based microfluidic systems for biosensing and sweat analysis. (a) A wearable plasmonic paperfluidic device that integrates sweat extraction and storage with rapid surface-enhanced Raman spectroscopy (SERS) analysis. Reproduced from ref. 143 with permission from American Association for the Advancement of Science, copyright 2022. (b) A wearable, paper-based chemiresistive sensor for monitoring human perspiration. Reproduced from ref. 146 with permission from Wiley-VCH, copyright 2019. (c) A multimodal, porous, soft bioelectronic system incorporating CNF-based microfluidics for monitoring energy metabolism and consumption. Reproduced from ref. 142 with permission from Wiley-VCH, copyright 2025.

A wearable chemiresistor featuring ink-printed carbon nanotube (CNT) patterns on conventional cellulose-based filter paper was developed to monitor sweat loss (Fig. 6b).^146^ The system incorporates an absorbent patch, a chemiresistor and a wireless reader to enable sweat monitoring on the body. Notably, the device exhibited a rapid response to perspiration during cycling exercise. A stable, planar signal during the first five minutes and a sharp signal change after 20 minutes corresponded to different phases of perspiration. Furthermore, the device effectively tracked the typical increase in sweat loss experienced during outdoor exercise.

Nevertheless, the capillary transport of biofluids from the skin to the sensing region is often impeded by the hydrophobic boundaries of the electrode patterns. A promising strategy to address this issue is the laser-induced pyrolysis of cellulose within paper substrates using a CO_2_ laser cutter/engraver. This technique enables the direct patterning of conductive traces and electrodes with well-defined geometries and tailored surface wettability on soft paper while preserving the capillary network structure.^130^ Inspired by this concept, a variety of flexible electronic circuits, mechanical sensors and biomolecular detection systems have been developed. Unlike laser-induced graphenization on polyimide films, which typically creates only electroactive electrodes, laser engraving of cellulose-based substrates enables the simultaneous fabrication of fluidic and electrical pathways. This dual functionality promotes the efficient transport of both biofluids and electrical signals. For instance, the Shih group reported on the fabrication of an electrochemical paper platform using laser-induced pyrolysis.^130^ First, electrochemically active electrodes and graphenic conductive traces were embedded within the paper substrate *via* laser engraving. Subsequently, fluidic channels were patterned onto the cellulose film using wax lamination. The resulting integrated device featured individually isolated and defined regions, enabling precise control over surface wettability, buffer flow and the electrochemical detection of HPV type 16.

Towards multimodal and closed-loop systems, integrating multimodal sensing with physiological feedback mechanisms is a critical frontier in wearable biosensing. However, customizing wearable bioelectronics requires the development of next-generation microfluidic biosensors with carefully designed channels to minimize signal interference and crosstalk. This improves the accuracy, reusability and robustness of multiplexed biosensor arrays. A recent study addressed this challenge by reporting a multimodal, porous, soft bioelectronic system incorporating cellulose nanofibril (CNF) interfaces for simultaneously monitoring electrocardiograms (ECGs), glucose and β-hydroxybutyrate (β-HB) in sweat—key biomarkers for managing energy metabolism and consumption (Fig. 6a). The integrated system features a multilayer architecture comprising a porous elastomeric substrate, sweat sensors, paper-based microfluidics, agarose hydrogel and ECG electrodes. Notably, the embedded CNF-based microfluidics enable on-demand sweat extraction and continuous refreshment, thereby enhancing the accuracy and consistency of biochemical sensing. The device was applied to the upper arm of a human subject and evaluated over a seven-day period (Fig. 6c). The CNF interfaces provided superior interfacial robustness, enabling the resilient integration of bioelectronic components on porous, soft substrates under strain. Stable ECG signals were continuously recorded throughout the week. Additionally, the system effectively captured changes in the composition of the sweat, with low glucose concentrations and elevated β-hydroxybutyrate (β-HB) levels being detected after 12 hours of fasting, indicating a shift towards ketone metabolism. Following the consumption of a keto drink, β-HB levels increased further while glucose levels decreased, subsequently rising upon carbohydrate intake.

Additionally, paper-based colorimetric sensors offer intrinsic visual readout and reagent-free operation, making them a compelling alternative for point-of-need applications. In thread–paper hybrid devices, hydrophilic threads guide sweat analytes towards colorimetric detection zones, enabling the simultaneous quantification of pH and lactate. These systems combine the affordability and breathability of fibrous substrates with reliable analytical performance, making them ideal for wearable biosensing in low-resource or ambulatory settings. In thread–paper hybrid devices, hydrophilic threads guide sweat analytes towards colorimetric detection zones, enabling simultaneous quantification of pH and lactate. These systems combine the affordability and breathability of fibrous substrates with reliable analytical performance, making them ideal for wearable biosensing in low-resource or ambulatory settings.

## Challenges and outlook

Paper-based microfluidic devices represent a transformative opportunity for wearable healthcare, offering an integrated combination of low cost, fabrication simplicity, and functional adaptability. Their intrinsic capillary-driven fluid handling, mechanical compliance, breathability, and compatibility with scalable manufacturing make them ideal for real-time diagnostics and continuous health monitoring. However, translating these devices from the laboratory to clinical and commercial use remains challenging and is still in its early stages.

### Technical challenges

Despite their significant potential, paper-based microfluidic devices are hindered by critical technical limitations that prevent their wider adoption. One of the main challenges is the variability of paper substrates, which makes it difficult to ensure reproducibility and batch-to-batch consistency due to differences in porosity, fiber orientation and surface hydrophilicity. Most existing platforms rely on colorimetric detection, which, while simple and equipment-free, typically offers a limited dynamic range, analytical precision and capability for multiplexing. Electrochemical sensors, which are essential for high-performance diagnostics, often disrupt capillary flow due to the hydrophobicity of conductive inks commonly used, which impedes fluid transport through the fibrous network. Therefore, innovative strategies are required to integrate electrodes into paper substrates in a manner that preserves capillary-driven flow whilst enhancing sensitivity and signal fidelity. Additionally, traditional fabrication methods such as wax printing have become increasingly impractical due to discontinued equipment and limited scalability. Although emerging techniques, including laser pyrolysis, nanocellulose-based inkjet patterning and photolithographic polymerization, show promise, they still require optimization and standardization for large-scale, cost-effective manufacturing.

### Clinical and translational challenges

From a clinical translation perspective, paper-based wearable devices face a range of physiological and system-level challenges. A critical challenge in the development of paper-based microfluidic devices is the sterilization of porous paper substrates. Given the potential for these devices to come into contact with various biofluids, such as interstitial fluid (ISF), saliva, and sweat, microbial contamination poses a significant health risk. The porous structure of paper can harbor microorganisms, making proper sterilization essential. Traditional sterilization methods, such as radiation, are often unsuitable because paper-based materials are typically non-transparent, complicating the sterilization process. Therefore, novel sterilization strategies must be developed to ensure the safety and efficacy of these devices in clinical settings. Additionally, the inherent porosity of paper introduces challenges related to fluid evaporation and the absorption of key analytes. Evaporation can result in sample volume loss, leading to inaccuracies in biofluid analysis, particularly in continuous monitoring scenarios. Furthermore, the absorption of critical components by the paper may alter analyte concentrations, affecting the precision and reliability of diagnostic results. Addressing these issues requires careful optimization of the paper's surface chemistry and porous structure, as well as the integration of measures to reduce evaporation, such as encapsulation or the incorporation of hydrogels to stabilize fluid volumes. These translational challenges must be addressed to enable the widespread adoption of paper-based microfluidic devices in point-of-care diagnostics. Improving sterilization methods, minimizing evaporation, and mitigating analyte absorption will be crucial for the successful clinical implementation of these devices.

Biofluids such as sweat and saliva are inherently dynamic, prone to evaporation, and subject to considerable intra- and inter-individual variability. Robust fluid handling mechanisms, such as chrono-sampling valves, capillary timers, and anti-biofouling interfaces, are essential to mitigate temporal artifacts, backflow contamination, and signal drift. In addition, the limited mechanical robustness and long-term durability of paper-based microfluidic materials, especially under wet conditions such as those encountered with biofluids like sweat and saliva, are significant challenges that need to be addressed to ensure reliable and precise wearable biosensing. Furthermore, user comfort further complicates the development of paper-based microfluidic devices, as wearables must maintain stable skin contact during motion without causing irritation, delamination, or material degradation. This highlights the importance of advanced paper-based materials engineering from the nanoscale, microscale to macroscale. Also, it is important to note that only a limited number of paper-based wearable devices have progressed beyond proof-of-concept studies. The absence of comprehensive clinical validation standardized analytical performance metrics, and regulatory alignment poses significant barriers to commercialization. To overcome these hurdles, design thinking, cost-of-goods modelling and target product profiling must be incorporated early in the development process to enable scalable, clinically relevant solutions.

### Future directions

The next phase of paper-based wearable microfluidics will focus on integrating advanced biosensing technologies, soft electronics and digital infrastructure. Incorporating molecular recognition elements, such as aptamers, antibodies and molecularly imprinted polymers, will improve the specificity of biomarkers and enable continuous, real-time monitoring. When coupled with electrochemical sensors, wireless data transmission capabilities, and machine learning algorithms, these platforms will support closed-loop feedback systems, facilitating dynamic health management, real-time feedback, and personalized intervention. In addition, advances in materials science will also play a pivotal role in future developments. Biodegradable, cellulose-derived nanostructures offer environmentally sustainable substrates that can be tuned for wetting behaviors, porosity and mechanical flexibility. The development of stretchable, multilayered, paper-based microfluidic paths and circuits will enable the integration of sampling, sensing and computational functionalities in a single, skin-conforming device.

## Conclusion

Paper-based microfluidics have emerged as a compelling platform for wearable soft bioelectronics, offering unique advantages in terms of mechanical compliance, breathability, capillary-driven flow, and cost-effectiveness. By enabling non-invasive sampling and real-time on-skin analysis of various biofluids including sweat, interstitial fluid, saliva, and wound exudate, bridging the gap between rigid laboratory diagnostics and personalized, real-time health monitoring, particularly in point-of-care settings. Nevertheless, clinical translation remains challenged by biofluid variability, limited sample volumes, and the instability of paper–electrode interfaces under dynamic physiological strain. Future research should prioritize the integration of advanced paperfluidic materials with real-time wireless communication and machine learning algorithms to drive progress toward next-generation, intelligent, closed-loop, point-of-care, and accessible diagnostic solutions.

## Author contributions

Feng Zhang: writing – review & editing, writing – original draft, visualization, conceptualization. Ganggang Zhao: visualization, validation. Qunle Ouyang: visualization, validation. Sicheng Chen: visualization, validation. Zheng Yan: writing – review & editing, writing – original draft, supervision, project administration, funding acquisition, conceptualization.

## Conflicts of interest

There are no conflicts to declare.

## Data Availability

No primary research results, software or code have been included and no new data were generated or analysed as part of this review.
